# Shockwaves Increase In Vitro Resilience of *Rhizopus oryzae* Biofilm under Amphotericin B Treatment

**DOI:** 10.3390/ijms23169226

**Published:** 2022-08-17

**Authors:** Cyrill Slezak, Karaleen Anderson, Tyson Hillock, Mariel Miller, Peter Dungel, Olga Kopp, Katja Sterflinger, Paul Slezak

**Affiliations:** 1 Department of Physics, Utah Valley University, Orem, UT 84058, USA; 2 Ludwig Boltzmann Institute for Traumatology, The Research Center in Cooperation with the AUVA, 1200 Vienna, Austria; 3 Austrian Cluster for Tissue Regeneration, 1200 Vienna, Austria; 4 Academy of Fine Art Vienna, 1010 Vienna, Austria

**Keywords:** shockwave therapy, biofilm, biophysical therapy, ultrasound, fibrin

## Abstract

Acoustical biophysical therapies, including ultrasound, radial pressure waves, and shockwaves, have been shown to harbor both a destructive and regenerative potential depending on physical treatment parameters. Despite the clinical relevance of fungal biofilms, little work exits comparing the efficacy of these modalities on the destruction of fungal biofilms. This study evaluates the impact of acoustical low-frequency ultrasound, radial pressure waves, and shockwaves on the viability and proliferation of in vitro *Rhizopus oryzae* biofilm under Amphotericin B induced apoptosis. In addition, the impact of a fibrin substrate in comparison with a traditional polystyrene well-plate one is explored. We found consistent, mechanically promoted increased Amphotericin B efficacy when treating the biofilm in conjunction with low frequency ultrasound and radial pressure waves. In contrast, shockwave induced effects of mechanotransduction results in a stronger resilience of the biofilm, which was evident by a marked increase in cellular viability, and was not observed in the other types of acoustical pressure waves. Our findings suggest that fungal biofilms not only provide another model for mechanistical investigations of the regenerative properties of shockwave therapies, but warrant future investigations into the clinical viability of the therapy.

## 1. Introduction

Extracorporeal shockwave treatment (ESWT) is an effective treatment strategy used to treat chronic soft tissue wounds, including burn wounds as well as diabetic and vascular ulcers [1]. While previous reports suggest that extracorporeal shockwaves at a sufficiently large energy flux density (EFD) are effective at causing damage to bacterial biofilms in vitro [2,3,4,5], the effect of shockwaves on fungal biofilms has been largely ignored.

Biofilms are defined as highly organized, surface associated communities of microorganisms that are protected within an extracellular matrix [6]. This protection provides an increased resistance to antimicrobial agents, with many species displaying up to 1000-fold resistance [7,8]. Many pathogenic bacteria and fungi can form biofilms in or on tissues, causing inflammation and tissue damage. Furthermore, planktonic bacteria released from these biofilms can cause chronic infections or infections at new sites away from the initial biofilm formation site [9].

Fungi belonging to the class Zygomycetes, namely *Rhizopus*, *Mucor*, and *Lichtheimia* (previously known as *Absidia*), have been implicated in an opportunistic, and sometimes fatal, infection known as Mucormycosis [10,11,12]. Found living ubiquitously in the environment, this class is usually associated with uncontrolled diabetes, diabetic ketoacidosis, hematological problems, malnutrition, trauma, and burns [10,11,12]. Amphotericin B (Amp B) has been used as the first line of treatment for Mucormycosis since the 1950s. However, it can have many adverse side effects, including chills, fever, headaches, loss of appetite, muscle pain, nausea, weight loss, and fatal syndromes of hepato and nephrotoxicity [13,14]. Despite the growing clinical significance of this disease, novel antifungal susceptibility testing on this species has remained largely uninvestigated [10,11,12].

Recent studies in culture-independent sequencing have shown a high prevalence of chronic wounds containing both fungal and bacterial species [15,16,17,18,19]. In studies of diabetic foot ulcers, polymicrobial biofilms containing fungal and bacterial species have been identified in over 75% of cases, associated frequently with *Staphylococcus aureus* and *Pseudomonas aeruginosa* [19,20,21]. Fungi have even been shown to have a commensal interaction with *Staphylococcus aureus*, promoting structural and chemical drug resistance. In vitro, in vivo, and clinical studies have shown that ultrasound treatments of 50 kHz−1 MHz disrupt both bacterial and fungal biofilms, specifically with *Staphylococcus aureus* and *Pseudomonas aeruginosa* bacteria [22,23,24,25]. It is therefore likely that the ultrasound treatment of polymicrobial biofilms would effectively neutralize both fungal and bacterial biofilms and facilitate drug delivery and efficacy.

The initial aim of this work was to test for improved drug efficacy through synergistic mechanical biofilm disruption when applying low frequency ultrasound (LFUS), radial pressure waves (RPW), or shockwaves (SW) on in vitro formed *Rhizopus oryzae* biofilms. An unanticipated increased Amp B resistance was observed when the biofilms were treated with shockwaves, which led to a subsequent investigation detailing the resulting biological response. Here, we investigate whether shockwaves are also stimulating in vitro cell proliferation and differentiation in fungi, similar to the effect seen in soft tissue and nerve regeneration studies [1,26,27,28].

## 2. Results

**Figure 1:**
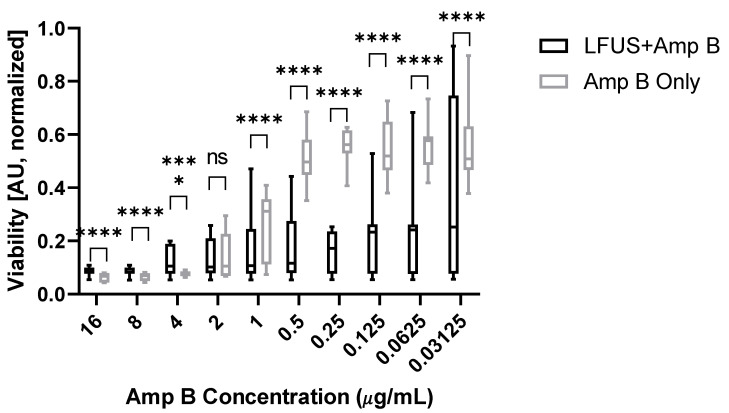
Effects of Amp B on the cell viability of *R. oryzae* biofilms with or without ultrasound treatment. This figure shows XTT absorption for varying concentrations of Amp B normalized to untreated controls. LFUS significantly enhances Amp B effects at concentrations of <1 µg/mL. (**** *p* > 0.0001).

Figure 1 shows a 12 h post-treatment viability comparison of the control biofilm normalized XTT absorption for varying concentrations of Amp-B-treated biofilms (n = 27 for each concentration), as well as those that received additional LFUS treatment (n = 54 for each concentration) at fixed LFUS treatment parameters of 550 kHz, 0.43 Vpp, 1 W/cm^2^. We found a high efficacy (>89%) of Amp B at high concentrentations > 1 μg/mL for destroying the biofilm. However, for lower concentrations, the Amp-B-only-treatment effectiveness wanes, although a large variability was observed over multiple runs. In contrast, a significantly lower (*p* > 0.0001) XTT absorption occured when LFUS was applied to the Amp-B-treated sample, indicating fewer remaining metabolically active cells, resulting in a less viable biofilm. The most significant difference was seen at Amp B concentrations of 1–0.031 μg/mL, where the efficacy of Amp B alone was 42–68%, while LFUS + Amp B remained > 74%. Additionally, mechanical disruption of the fungal biofilms was seen in the LFUS controls without Amp B; however, this was not observed in the biofilm controls (no Amp B + no LFUS), which was confirmed with additional replications.

**Figure 2:**
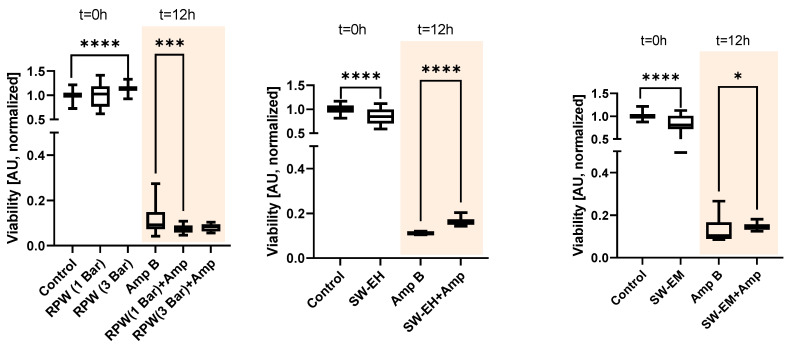
Immediate acute (t = 0) and longitudinal (t = 12 h) effects of Amp B with or without acoustical pressure wave treatment on *R. oryzae* biofilms. The figure shows normalized XTT absorption for healthy and Amp-B-treated (8 μg/mL in 1% DMSO) biofilm when treated with pressure waves/shockwaves. While radial pressure waves (**left**) had a synergistic effect on Amp B efficacy (AU 0.11 → 0.07 and →0.08 for 1 and 3 bar, respectively), both SW-EC (**center**, AU 0.11 → 0.17) and SW-EM (**right**, AU 0.10 → 0.14) prevented Amp B-mediated cell death after 12 h (* *p* > 0.05. *** *p* > 0.001 **** *p* > 0.0001).

A similar effect could be observed when the samples were treated with radial pressure waves (RPW). Figure 2, left, shows the results for two different energy settings of the device with 1 and 3 bar of air pressure, respectively, applied to the ballistic projectile. In the treatment of an untreated biofilm with radial pressure waves, a slight immediate (t = 0 h) increase in the median normalized XTT absorption of the higher energy waves at 3 bar (+13%, n = 36, H(2) = 38.71, *p* > 0.0001) over the biofilm control group (n = 162) was seen, but no statistical difference was found for the 1 bar treatment (n = 45, *p* > 0.604). When comparing the two treatment groups to the Amp B only control (n = 180) after 12 h, we found a significantly (H(2) = 15.70, *p* > 0.0004) lower absorption of 23% for the 1 bar group (n = 45), while no significance was found in the 3 bar group (n = 36, *p* > 0.705).

Next, shockwaves, which are short lifecycle acoustical pulses characterized by high peak pressure with steep pressure gradients, were investigated. The application of both electrohydraulic shockwaves (SW-EH, EFD = 0.19 mJ/mm^2^, Figure 2, center) and electromagnetic shockwaves (SW-EM, EFD = 0.12 mJ/mm^2^, Figure 2, right) to the samples yielded the exact opposite behavior. We first observed an initial, immediate (t = 0 h) slight destructive behavior for both types of applied shockwaves to the untreated biofilm, as indicated by the significantly decreased viability median absorption values (−16%, U = 1139, *p* > 0.0001 and −19%, U = 1237, *p* > 0.0001). Here, all group sizes were n = 72.

Second, in stark contrast with the previously observed Amp B enhancing results for LFUS and RPW, the application of EH and EM shockwaves to the Amp-B-treated biofilm resulted in a marked statistically significant lower Amp B efficacy. This was indicated by the biofilm’s increased viability, as indicated by the larger median normalized XTT absorption over the Amp B control (+49%, U = 0, *p* > 0.0001 and +36%, U = 1300, *p* > 0.025) after 12 h, suggesting a distinct regenerative/protective effect of these shockwaves.

**Figure 3:**
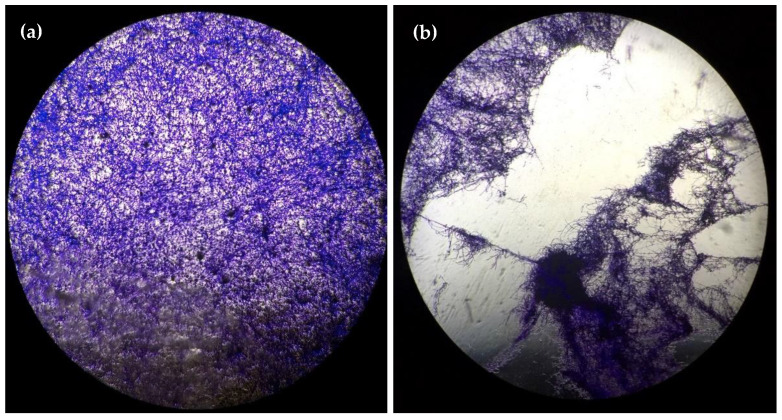

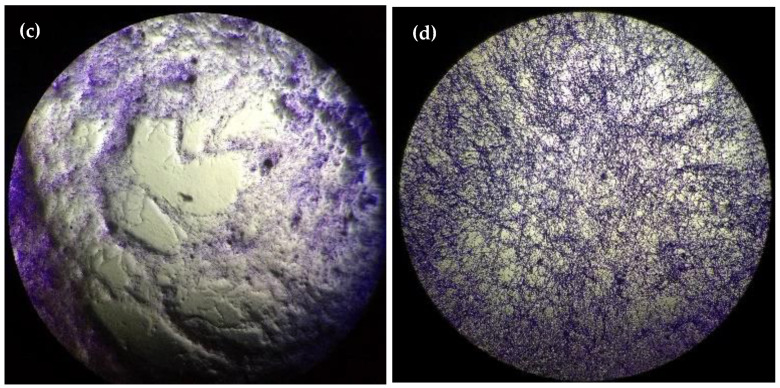
Microscopy images at 4× magnification of well-plate grown biofilms using crystal violet staining under different treatment conditions showing the (**a**) untreated biofilm control, (**b**) mechanically shred biofilm immediately after high-energy (0.55 mJ/mm^2^) EM shockwave treatment, (**c**) Amp-B-treated biofilm after 12 h, and (**d**) combination of Amp B + medium energy (0.19 mJ/mm^2^) EH shockwave treatment after 12 h.

Figure 3 visually summarizes the clear impact of the shockwave treatment on the overall structure of the biofilm. When treating a healthy biofilm (Figure 3a) with high-energy (EFD = 0.55 mJ/mm^2^) EM shockwaves, applied in the vicinity of the air interface, we observed immediate (t = 0 h) tearing of the biofilm, which was evident by the frayed edges (Figure 3b). In contrast, treatment with Amp B resulted in spread-out continual damage to the biofilm marked by soft transitions (Figure 3c) after 12 h post-treatment (t = 12 h). Finally, the additional medium-energy (EFD = 0.19 mJ/mm^2^) EH shockwave induced biological response of the Amp-B-treated biofilm resulted in substantial recovery of the overall structure and integrity (Figure 3d) over the same period.

**Figure 4:**
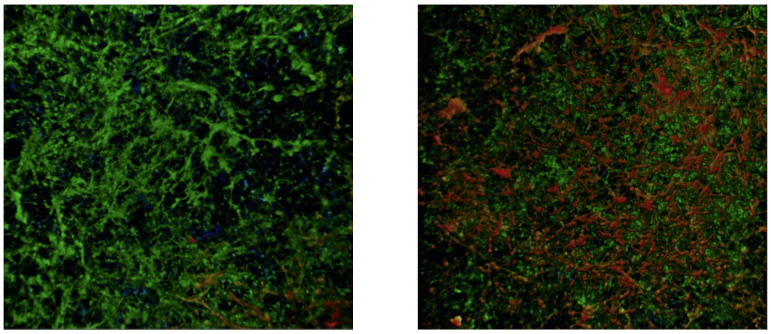
Confocal microscopy images at 10× magnification of the Amp-B-only-treated biofilm (**left**) and the biofilm treated with Amp B + electrohydraulic shockwaves (**right**) using calcofluor white staining. Qualitative comparison using false color enhanced red indicating deeper, fuller ECM, and biofilm for the shockwave sample.

In addition to cellular viability and the subsequently discussed proliferation, there was a distinctive impact on the extracellular matrix (ECM) structure. The representative confocal microscopy images in Figure 4 revealed a greater depth and increased ECM in the shockwave + Amp-B-treated biofilm compared with the Amp-B-only-treated ones 12 h post-treatment.

**Figure 5:**
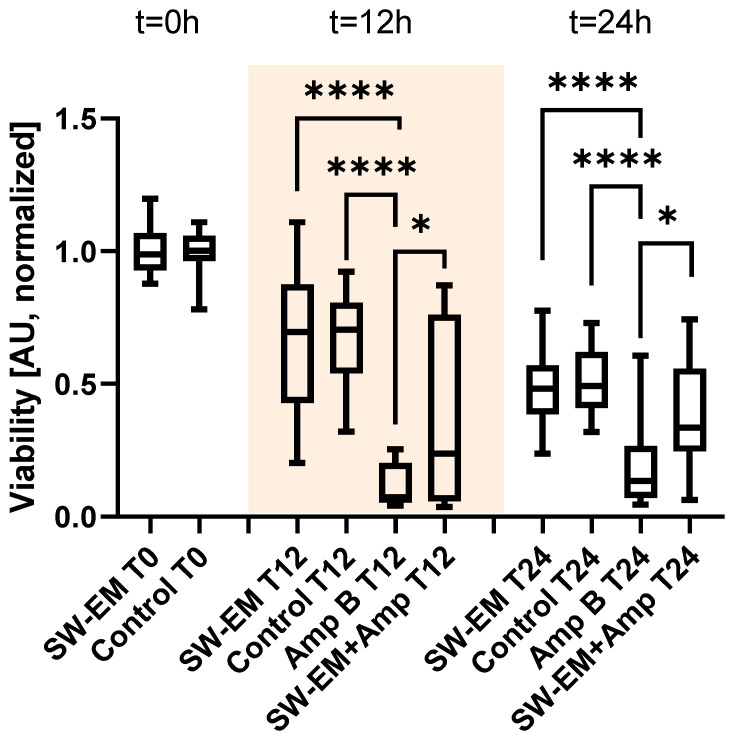
Effects of Amp B on cell viability with or without electromagnetic shockwave (SW-EM) treatment on *R. oryzae* biofilms grown on a fibrin matrix. The figure shows normalized XTT absorption for healthy and Amp-B-treated biofilms at timepoints T0 h, T12 h, and T24 h. There is a marked increased viability for the shockwave-treated biofilm over the Amp B only group after both 12 h (AU 0.07 → 0.24) and 24 h (AU 0.13 → 0.34). (* *p* > 0.05, **** *p* > 0.0001).

In a subsequent step, in order to better approximate the basic wound morphology, the fungal biofilm was grown on a fibrin matrix substrate and the proximity of the inherent air interface of the well-model was removed by using filled falcon tubes. The biofilm was treated with an electromagnetic shockwave applicator (EFD = 0.55 mJ/mm^2^) and readouts were taken immediately after the treatment (t = 0 h), 12 h post-treatment (t = 12 h), and an additional longitudinal 12 h thereafter (t = 24 h) (see Figure 5).

There was no apparent effect of the shockwaves on the healthy biofilm cellular viability immediately after or subsequent to the treatment after the proximity of the air interface was removed from the model. At t = 0 h, there was no statistically significant difference (U = 434, *p* > 0.820) between the XTT absorption of the untreated biofilm control (n = 30) and the one treated with shockwaves (n = 30). The same trend was observed for the untreated biofilm and the shockwave-treated one, as no statistically significant difference was found at times t = 12 h (H(3) = 59.63, *p* > 0.999) and t = 24 h (H(3) = 44.16, *p* > 0.999) for a sample size of n = 33 for each group.

Comparing the individual groups to the Amp B control group (n = 27) after 12 h, we detected a significant (*p* > 0.0001) impact of the antifungal medication resulting in very low XTT absorption. However, similar to the previously discussed plate-treated results, we observed that the additional shockwave treatment (n = 27) resulted in a significant (*p* > 0.0106) increase of 220% viability over the Amp B control.

After 24 h, the untreated and shockwave-only-treated biofilm viabilities continued to decrease to their lowest values. In contrast, both Amp-B-treated groups showed higher XTT absorption values, but the significant (*p* > 0.0116) diminutive effect of the applied shockwaves of the Amp-B-treated samples remained as indicated by a 149% increased viability over the Amp B control.

**Figure 6:**
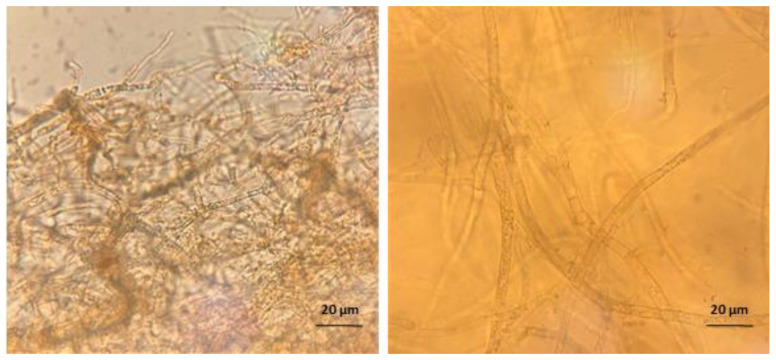
Microscopic images at 10× magnification showing hyphal width: qualitative comparison of biofilm grown on plastic (**left**) and biofilm grown on fibrin (**right**).

The biofilms grown on fibrin substrates behaved similarly to their well-plate counterparts when exposed to either Amp B, shockwaves, or a combination thereof. Figure 6 shows a close-up comparison of a microscopy image taken from two biofilms grown on plastic or fibrin.

**Figure 7:**
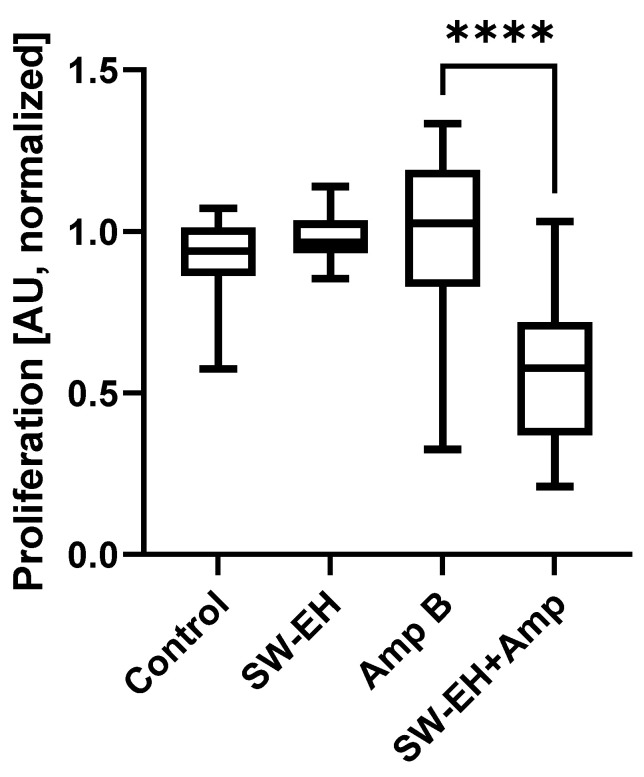
Effects of Amp B with or without SW-EH treatment on the proliferation of *R. oryzae* grown on a fibrin matrix 12 h post-treatment, showing a significant drop in the combined treatment’s proliferation over the Amp B one (AU 1.0 → 0.58). Proliferation was quantified using the BrdU assay (**** *p* > 0.0001).

In order to gain a better understanding of the impact of shockwaves on the biofilms and to better separate the effects on cellular viability and proliferation, a BrdU assay was performed on the fibrin substrate group. Figure 7 shows the results for both the untreated groups (n = 36 each) and the two Amp-B-treated groups (n = 72 each). There was no statistically significant difference (H(3) = 99.49) between the Amp B control group and the healthy biofilm (*p* > 0.134) or the shockwave group (*p* > 0.999). However, there was a significantly (*p* > 0.0001) lower absorption in the Amp B + shockwave-treated group. 

## 3. Discussion

In the biofilm viability results, a distinctively different behavior can be seen between the ultrasound and radial pressure waves on the one hand, and shockwaves on the other. The nature of the underlying physical stimuli differs in various wave parameters, including energy flux density, peak pressure, and pressure gradient, resulting in different biological responses.

In addition to mechanotransduction-initiated biological responses, there is evidence of purely mechanical effects. When applying SW-EM to the biofilm inside the well plates at 0.55 mJ/mm^2^, the biofilm is visibly shredded by the pressure waves. The application of large mechanical sheer/stretch forces on the biofilm leads to a disruption of the hyphae structure, resulting in a disintegration of the biofilm when applying high-pressure shockwaves. This is associated with the tensile part of the wave pulse, which is amplified when the pulse reflects at a fluid−air interface in the vicinity of the to-be-treated sample [29]. A similar, yet smaller destructive effect can be seen on the biofilm when exposed to lower-pressure shockwaves, as evidenced by a lower cellular viability immediately after treatment. In contrast, no such effect was observed in the fully submerged tube samples for all of the studied energies, underlining the impact of air interfaces in the vicinity of the treatment zone.

The synergistically improved fungicidal efficacy of Amp B in combination with LFUS and RPW can be attributed to the additional distribution and mixing of the medication throughout the biofilm matrix, thus enhancing the proposed extraction of ergosterol from the lipid bilayer [30]. This process may be driven by the presence and creation of microbubbles during treatment. In our experiments, acoustic cavitation could occur in ultrasound and radial pressure wave treatments, while the applied shockwaves also had sufficient tensile pressure to trigger additional inertial cavitation bubbles [31]. This is supported by the fact that the application of LFUS to bacterial biofilms has been shown to significantly impact their viability and morphology [32], associated with non-inertial cavitation associated with trapped microbubbles [33].

Additional potential thermal effects [32] are unlikely candidates to affect the viability of the biofilm in our experiments. Thermal heating is most often associated with ultrasound treatments due to high frequencies and long duty cycles. As the pulse rate is of only a few Hz in ESWT, heating was only a potential concern in our LFUS treatment. Using a limited 50% duty cycle at low intensities resulted in no expectation of local heating [32]. In addition, there was a significantly reduced thermal power deposition of subsequent acoustical pulses when cavitation-driven bubble fields were encountered [34].

Finally, we observed no tearing or shredding, as well as no acute and imminent (t = 0 h) impact on the viability or proliferation of shockwave-only-treated biofilms in the absence of Amp B in the fibrin model where the air interface was removed. It was expected that during synergistic shockwave treatment, mechanical agitation and cavitation enhanced Amp B efficacy also existed at later timepoints (t = 12 h and t = 24 h), but this was covered up by the inseparable additional stronger protective biological response, which was expectedly larger than that which was experimentally observed in our system.

Two methods were used for biofilm growth, a well-plate method, as previously performed by Pierce et al. [35], and a fibrin substrate method for optimal shockwave delivery. Biofilm substrates have proven to be an integral factor in biofilm development, specifically the role played by collagen, fibrinogen, and fibrin in chronic wound biofilms [36,37,38,39]. Creating a scaffolding using fibrin glue and fibrin-coated surfaces is a common method for in vitro models of chronic wounds [40,41], which was adapted here. Additionally, fibrin-binding mechanisms have been identified in several fungal species and are likely contributing factors for improved biofilm structural development on fibrin substrates [39,42]. We indeed found a clear structural difference between the two biofilms’ hyphal width, as can be seen in Figure 6. While this could impact some of the mechanical factors discussed in the previous section, it is unlikely that we would see a fundamentally different trend due to the overall length scales in comparison with the acoustical wavelengths.

A controlled structural model is important, as previous studies have shown that the mechanical impact of shockwaves on the ECM increases the release of exosomes and the regeneration of ischemic myocardium [43,44]. The production and release of these extracellular vesicles (EVs) are associated with various mechanical stressors imparted on the cells [45]. EVs have also been described in *Rhizopus* species and pathogen-derived exRNAs can be used as biomarkers of infection as they can be detected in the body fluids of patients with mucormycosis [46]. It has been postulated that these extracellular vesicles are involved in intercellular exchange of information, resulting in cell activation and growth [47,48]. It is possible that this mechanism was also involved in the structure and increased growth of *R. oryzae* in our experiment.

Mechanical disruption of the biofilms using LFUS and RPW led to a significantly enhanced Amp B efficacy in our in vitro model. By extrapolating from our experiment to an in vivo system, we expect to see the potential for greater clearance by host innate and adaptive immune cells positioning LFSU as a potentially non-invasive and non-toxic synergistic treatment to Amp B against Mucormycosis infections.

In contrast, in vitro shockwave-treated cells, while arguably also subject to the agitation-enhanced Amp B effectiveness, shows an overall increased viability. This is especially remarkable in light of the mechanical Amp B enhancing factors discussed earlier. Increased viability and the overall regenerative potential of shockwaves have previously also been demonstrated in bacteria and mammalian cells [49]. Observing a similar behavior in fungi, as shown in this study, further supports a common mechanism.

Previous works provide additional insight into the possible working mechanisms. Membranes are sensitive to mechanical forces and shockwaves have been shown to change membrane permeability through the forces induced, which result in relative motion [50]. An analysis of the changes in the cell membrane associated with shockwaves indicates a non-uniform pressure distribution that creates pores on the membrane. This depends on the profile of the shockwave: shorter pulse widths result in the formation of pores and larger ones cause compression of the cytoplasm, limiting the formation of pores in the membrane and therefore preserving the cell [51,52]. The cell wall of fungi is essential for their growth and plays an important role in the interaction of the fungi with the cells of the immune system [53,54]. The cell wall changes under environmental or cellular stress [54]. In some fungi, such as *Antrodia cinnamomea*, ultrasound damages the cell wall of spores, allowing for easier spore germination [51] in the absence of antifungal treatment.

Considering the interplay of the observed results of increased cellular viability and proliferation requires some additional context. It has been reported that when using biophysical stimuli, data of the proliferation and metabolic activity are not directly correlated. In general, the BrdU assay is more sensitive, and a cellular response can be perceived earlier than with XTT. Hence, the effect seen in BrdU is not necessarily reflected in the XTT results.

In a recent publication, Balzer et al. [55] showed that an increase in cell activity by a resazurin-based assay was associated with a decrease in Hoechst-stained nuclei counts. In addition, it is known that differences in the compound mechanisms of action and cell-specific responses can yield significantly misleading results when using ATP or tetrazolium-reduction assays. Quent et al. [56] reported on the discrepancies between the metabolic activity and DNA content, and concluded that metabolic assays may not accurately reflect the cellular proliferation rates due to a miscorrelation of metabolic activity and cell number.

## 4. Materials and Methods

### 4.1. Cell Culture

All LFSU experiments were performed at a laboratory at Utah Valley University, Orem, UT, USA, and all RPW and SW experiments were performed at the VIBT-Extremophile Center, University of Natural Resources and Life Sciences (BOKU), Vienna, Austria, using a second strain of *R. oryzae*. The ultrasound experiments performed in the US used *R. oryzae* (ATCC 34965) and the shockwave experiments in Austria used *R. oryzae* (strain ZM18), provided by the BOKU. Identical methods were used by the same team of scientists at both locations. Lyophilized *R. oryzae* was re-suspended in 0.2–0.5 mL of sterile water for 30 min. The resulting suspension was inoculated onto potato dextrose agar (PDA) (Sigma-Aldrich, Saint Louis, MO, USA) and left to grow at room temperature for 4 days. Following the initial growth, all cultures were continually transferred onto PDA for 4–7 days before harvesting. The cells were harvested by flooding the cultures with 10 mL PBS/Tween solution (Sigma-Aldrich, Saint Louis, MO, USA). This suspension was then vortexed and centrifuged at 3000 rpm at 4 °C for 5 min. The resulting pellet was washed twice with sterile PBS, re-suspended in 10 mL pre-warmed RPMI-1640, and the spores were counted using a Neubauer Hemocytometer. The cell density was adjusted to 1 × 10^5^ cells/mL and suspended in RPMI-1640 (Corning-Cellgro, Manassas, VA, USA), without sodium bicarbonate, supplemented with L-glutamine and buffered with 165 mM morpholinepropanesulfonic acid (Sigma-Aldrich, Saint Louis, MO, USA).

### 4.2. Biofilm Formation

Following the protocols developed by Pierce et al. [35], the biofilms for LFUS treatment were grown in a 96-well Corning Costar flat bottom cell culture plate (Corning 3595, Corning Incorporated, Life Sciences, Kennebunk, ME, USA), and 100 μL of the 1 × 10^5^ cell/mL suspension detailed above was added to each well [35]. Biofilms for RPW and the shockwave treatments on plates were grown on a 24-well Corning Costar flat bottom cell culture plate (Corning 3473, Corning Incorporated—Life Sciences, Kennebunk, ME, USA), and 400 μL of the 1 × 10^5^ cell/mL suspension detailed above was added to each well.

Biofilms were also formed in 15 mL polypropylene tubes (Sigma-Aldrich, Saint Louis, MO, USA). Briefly, 0.5 mL of fibrin glue (Tisseel Fibrin Sealant, Baxter, Deerfield, IL, USA) was poured and allowed to set for 10 min before the addition of 400 μL of the cell suspension, so as to achieve 1× 10^5^ cells/mL suspension.

All of the biofilms, plated and in polypropylene tubes, were placed in an orbital shaker at 75 rpm and were incubated at 37 °C for 24 h. The resulting biofilms were washed three times with sterile PBS (Gibco, Grand Island, NY, USA) and aspirated prior to treatment.

### 4.3. Ultrasound and Antifungal Treatment

After washing, the biofilms were re-suspended in 100 μL RPMI-1640 (Corning-Cellgro, Manassas, VA, USA) and treated under four main conditions: LFUS only, LFUS + Amp B, Amp B only, and untreated Biofilm control (no Amp B, no ultrasound). The biofilms treated with ultrasound were placed on a piezoceramic ultrasonic transducer (TRS Technologies State College, PA, USA) matching the inner dimensions of a 96-well culture plate and were connected via a glycerin coupling gel. LFUS was generated with a 420 dual phase lock-in amplifier from Scitec Instruments (Trowbirdge, Wiltshire, UK). The LFUS parameters were 550 kHz, 0.43 Vpp, 1 W/cm^2^, on 50% duty cycle, and 10 min duration. Upon completion of the LFUS treatment, all biofilms were incubated in an orbital shaker with the same settings.

The Amp B solution was made by preparing two-fold serial dilutions of Amp B in 1% DMSO (Fisher BioReagents, Fisher Scientific, Fair Lawn, NJ, USA), after which 2% RPMI-1640 culture media was added to the ten Amp B dilutions. Each well received 100 μL of the corresponding Amp B solution (final concentration 16–0.03 μg/mL) or the DMSO + 2% RPMI control solution, and each treatment concentration was tested in triplicate. The plates were covered, sealed with Parafilm, and incubated in an orbital shaker at 75 rpm for 12 h at 37 °C.

### 4.4. Shockwave or RPW and Antifungal Treatment

Following incubation, the resulting biofilms were washed three times with sterile PBS and carefully aspirated. The plated biofilms were re-bedded in 400 μL RPMI-1640 and treated under four main conditions: shockwave or RPW only, shockwave or RPW + Amp B, Amp B control, and biofilm control (no Amp B, no shockwave or RPW). Mature biofilms grown on fibrin in falcon tubes undergoing treatment in the water bath were bedded in 5 mL RPMI-1640 to remove the air interface in the local vicinity of the sample and were treated under the same four conditions. Amp B was used at a concentration of 8 μg/mL in 1% DMSO (previously determined MIC_50_) for the plated and falcon tube biofilms.

Shockwaves were applied using an electrohydraulic (OP 155 connected to an Orthogold 100 device, MTS Medical, Konstanz, Germany) or an electromagnetic (DUOLITH SD1 «ultra» with the “Sepia” handpiece, Storz Medical, Tägerwilen, Switzerland) device. RPW were applied using DUOLITH SD1 «ultra» with the “FALCON” handpiece (Storz Medical, Tägerwilen, Switzerland). Pressure pulses were applied to the fully matured biofilm directly following the addition of Amp B. XTT readouts were taken immediately after (0 h), as well as 12 and 24 h post-treatment.

Plated biofilms were treated vertically through the bottom of the well-plate. The shockwave transducers were positioned so that the biofilm was located at the focal length of the device by adjusting the membrane inflation (EH) or using a focusing spacer (EM). The applicators were then coupled to the well-plates using ultrasound gel, assuring that the contact area was restricted to a single well-bottom. Soft electrohydraulic and electromagnetic applicator membranes had direct contact with the plates while the metallic RPW applicator was only coupled using gap-filling ultrasound gel but removing any direct physical contact. This was to remove any sonic components of the RPW applicator, thus only transmitting the ultrasonic waves. Sample wells were spatially separated on the plates and all nearest-neighbor wells, as well as interstitial spaces, remained air-filled to avoid double-treatment. Electrohydraulic treatment received 300 pulses using an energy density of 0.19 mJ/mm^2^ at 3 Hz. Electromagnetic treatment received 300 pulses using an energy density of 0.12 mJ/mm^2^ at 3 Hz. Radial treatment received 300 pulses at 1 Bar or 3 Bar.

**Figure 8:**
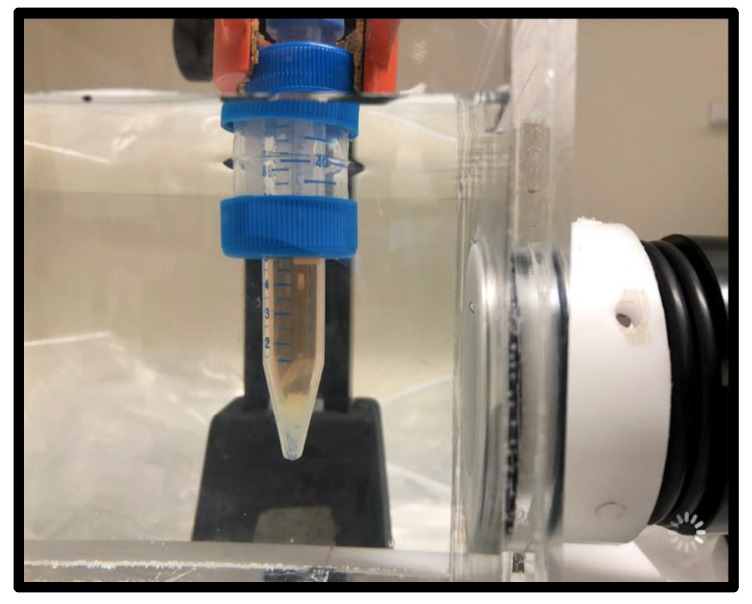
Electromagnetic shockwave applicator attached to the right side of a water bath treating a biofilm specimen inside the falcon tube. The substrate fibrin clot is visible at the bottom tip of the container, which is RPMI filled, removing air interfaces near the treatment zone.

Tubes containing the fibrin substrate biofilm (see Figure 8) were treated with 300 EM pulses using an energy density of 0.55 mJ/mm^2^ at 3 Hz. To allow for the unhampered physical propagation and standardized application of shockwaves to the sample, shockwave treatment was performed using a water bath setup [57]. Hydrophone guidance was used to place the biofilms inside the focal zone of the applicator for treatment. Then, 5 mL of RPMI with or without Amp B treatment was added to freshly aspirated mature biofilm in a 15 mL polypropylene tube and exposed to shockwaves under uniform and reproducible treatment conditions in terms of temperature and distance to the shockwave applicator. Control tubes were treated identically, but received no shockwave treatment, by adding them to the water bath and allowing them to sit for 2 min without being shocked.

### 4.5. Biofilm Metabolic Assay

Biofilm metabolic quantification for viability was done by treating with 400 μL (200 μL for LFUS) of an XTT (Biotium, Fremont, CA, USA) sodium salt/Menadione (MP Biomedical, Solon, OH, USA) solution to measure the ability of the metabolically active sessile cells to reduce the XTT to a water-soluble orange formazan compound [35,58]. The plates were covered in aluminum foil and incubated in the dark for 2–3 h at 37 °C. After incubation, three 100 μL samples (one 150 μL sample for LFUS) of the resulting colored supernatant were removed and transferred into the wells of a new microtiter plate. Each plate was then read in the BioTek Synergy H1 microplate reader at 490 nm (Agilent, Santa Clara, CA, USA). The resulting absorption values were normalized to the corresponding experimental biofilm control group.

### 4.6. Biofilm Cell Proliferation Assay

Cell proliferation was assessed using a BrdU labeling assay. BrdU (Bromodeoxyuridine/5-bromo-2’-deoxyuridine) is an analog of the nucleoside thymidine and is used to identify proliferating cells. During the BrdU assay, BrdU is incorporated into replicating DNA and can be detected using anti-BrdU antibodies. A 10 mM stock solution of BrdU was made by dissolving 3 mg of BrdU in 1 mL of water. This 10 mM BrdU stock solution was then diluted in RPMI-1640 to make a 10 μM BrdU labeling solution. A previous culture medium was removed from the biofilms and replaced with the 10 μM labeling solution and allowed to incubate for 12 h at 37 °C. Following incubation, BrdU labeling was removed, and the biofilms were washed three times with PBS. DNA hydrolysis was done by incubating cells in 1–2.5 M HCI for 1 h at room temperature and washing three times in PBS. The biofilms were then fixed according to standard immunocytochemistry protocols.

### 4.7. Microscopy

A Confocal Microscope (Leica TCS SP5, Scanning head SP5II with Acoustico-optical Beam Splitter and resonant scanner, Software FRAP Wizard, Leica Microsystems, Wetzlar, Germany) was used to view the overall structural integrity of the biofilms. Prior to microscopy, the biofilms were grown and treated as previously stated. Biofilms were stained using Calcofluor White (Megazyme C-CLFR, Ayrshire, Scotland) stain targeting the chitin in the cell walls and hyphae. Then, 200 μL of stock solution A (10 g KOH in 90 mL water and 10 mL glycerin) and 200 μL of stock solution B (0.1 g calcofluor white in 100 mL water) were added to each tube and placed in dark for 5 min. The biofilm was carefully transferred to a plate for microscopy. Additional standard crystal violet (CV) procedures were performed on selected samples. The biofilms were fixated with 400 μL of 99% methanol for 15 min before supernatant was removed and air dried. Then, 400 μL of 0.1% W/V CV was added to each well for 20 min at room temperature and then washed three times with PBS solution.

### 4.8. Statistics

Statistical two-sample comparisons used the Mann–Whitney U test and all multi-group statistical analyses were done using non-parametric Kruskal–Wallis tests, as not all groups passed normality testing. Additional group comparisons used Dunn’s multiple comparison test. The threshold value for statistical significance was chosen as α = 0.05 throughout. Experimental raw data sample sizes are indicated (n), including triplicate samplings (single sampling but triplicate readout for LFUS), before being cleaned of outliers using robust regression and outlier removal (ROUT) [59] with a coefficient Q = 1%. The box-and-whisker plots shown utilize a min-to-max whisker length.

## 5. Conclusions

This study provides clear evidence of low frequency ultrasound and radial pressure waves enhancing the effectiveness of Amphotericin B through synergistic mechanical agitation when treating in vitro *Rhizopus oryzae* biofilm. In contrast, the application of shockwaves resulted in a strong biological response of the biofilm, significantly increasing cellular viability in vitro, thus lowering the anti-fungal efficacy of Amphotericin B. Finally, establishing a fibrin-substrate-based biofilm model for the evaluation of shockwave therapies provided a reliable standardized model mimicking the basic wound morphologies and removing the air-interfaces as encountered in traditional polystyrene well-plate models. These findings further support the regenerative potential of shockwaves by illustrating that this effect exists even in fungal biofilms.

## Data Availability

All data and analyses are available upon request from the corresponding author.
